# The role and advantages of intraductal ultrasound for the management of gallstones

**DOI:** 10.2478/abm-2026-0001

**Published:** 2026-04-30

**Authors:** Editorial Office of Asian Biomedicine

**Affiliations:** Faculty of Medicine, Chulalongkorn University, Bangkok 10330, Thailand

Gallstones are a common reason for admission to the surgical service. In general, gallstones are located in the gallbladder of the biliary tree. Most stones consist of cholesterol from ingested food. Most people with gallstones do not have significant symptoms. A small percentage develop complications resulting in abdominal pain and other serious complications. Risk factors for gallstones and gallstone-related disease include female gender, obesity, and rapid weight loss. Most patients with gallstones do not have any symptoms, but some have symptoms requiring surgical interventions [1]. Regular physical activity and an appropriate diet are two of the most important measures for the prevention of gallstone-related disease [2].

Concomitant gallbladder and common bile duct (CBD) stones, known as cholecystocholedocholithiasis, are clinically prevalent. Endoscopic Retrograde Cholangiopancreatography (ERCP) is commonly used for the management of choledocholithiasis. ERCP is minimally invasive but may be associated with significant adverse events, particularly post-ERCP pancreatitis [3,4,5]. Moreover, small stones may be missing. Sometimes, it is difficult to differentiate stones from air bubbles, tumors, or clots. Furthermore, ERCP may reveal an inconclusive cholangiogram in about 10%–15% of cases.

Intraductal ultrasound (IDUS) is a sophisticated adjunctive technique that enhances the precision and success of ERCP for choledocholithiasis [6]. IDUS involves passing a miniaturized ultrasound probe (typically 2.0–2.4 mm in diameter) directly into the bile duct. It provides high-resolution, 360° cross-sectional, real-time images of the bile duct wall layers and surrounding structures (portal vein, hepatic artery, pancreas). The American Society for Gastrointestinal Endoscopy (ASGE) Standard of Practice (SOP) Guideline provides evidence-based recommendations for the endoscopic evaluation and treatment of choledocholithiasis [6].

In this issue, Liang et al. [7] reported the clinical value of IDUS in endoscopic extraction of choledochal stones. Drawing on experience from Asia, the authors describe the application of IDUS in both the diagnosis and management of choledocholithiasis [7]. They conclude that IDUS is particularly effective in detecting small stones (<3 mm) that are difficult to visualize with conventional cholangiography and that its use in ERCP combined with endoscopic sphincterotomy may reduce postoperative stone recurrence [7].

They emphasized that IDUS adds significant value to endoscopic retrograde cholangiography, particularly when incorporated into a planned therapeutic intervention [7]. However, many new techniques have been developed for optimal management of CBD stones. For example, while IDUS may be very useful in identifying small stones in the main duct, recent techniques such as cholangioscopy may offer direct visualization for precise management, especially in the intrahepatic ducts [8]. While IDUS provides excellent high-resolution imaging, cholangioscopy allows direct visual confirmation, useful for optimal intervention. Artificial intelligence also provides more potential applications in diagnostic imaging interpretation, intraprocedural decision support, and prediction of stone recurrence.

In conclusion, while IDUS has advanced our diagnostic and therapeutic management of CBD stones, new techniques have emerged today. With available options, more patient-specific, resource-sensitive approaches are possible. The integration of cutting-edge technologies and data-driven strategies can improve the overall quality of endoscopic CBD stone management. Modern digital cholangioscopes have improved their practicality, overcoming limitations of older systems [8].
